# Comparative Study of Blood, Tissue and Serum Levels of Carcinoembryonic Antigen (CEA) Detection in Breast Cancer

**DOI:** 10.31557/APJCP.2019.20.10.2979

**Published:** 2019

**Authors:** Neda Moazzezy, Saeid Bouzari, Mana Oloomi

**Affiliations:** *Molecular Biology Unit, Pasteur Institute of Iran, Tehran, Iran. *

**Keywords:** CEA, RT-PCR, breast cancer, prognosis

## Abstract

**Background:**

Carcinoembryonic antigen (CEA) detection was evaluated in breast cancer (BC). The statistical correlation between the CEA mRNA and clinico-pathological features in the peripheral blood (PB) and tissue samples of BC was assessed.

**Materials and Methods:**

RT-PCR (Reverse transcription-polymerase chain reaction) analysis was applied to study the expression of CEA in PB of 30 healthy females and 30 patients with operable BC before receiving any therapy, as well as in the tissue of 30 BC patients.

**Results:**

CEA was observed in a number of normal subjects, but there was a significant difference between the patients and controls. The detected CEA mRNA from tissue samples were the same as PB of patients and a correlation was observed between the CEA mRNA in PB and tissue samples (Pearson chi-square = 8.62, P=0.003). In the PB, CEA mRNA was significantly different in HER-2 (-)/HR (+) compare with HER-2(+)/HR (-) tumor group (p=0.026). Finally, CEA in serum was also significantly different in HER-2(-)/HR (+) compared with HER-2(+)/HR (+) and HER-2(+)/HR (-) subtypes (p=0.008 and p=0.043, respectively).

**Conclusion:**

CEA mRNA evaluation is diagnostically valuable as a breast cancer marker. Additionally, CEA can significantly improve the sensitivity of diagnosis.

## Introduction

As a heterogeneous disease and world-wide public health difficulty, breast cancer (BC) has the first rank among cancers diagnosed and fifth major cause of death in Iranian women (Farhood et al., 2018). Early detection of BC is one of the most beneficial and effective methods to reduce BC burden and mortality that can greatly increase the chances of effective treatment (Torre et al., 2017). Early cancer detection and screening have been enhanced by cancer biomarkers or tumor markers; which may facilitate high speed, non-invasive cancer diagnosis; the routes for translating new information into sensitive and specific diagnostic, prognostic and predictive tests are still being developed. A biomarker (biological marker) refers to “any measurable substance, structure or process in the body or biological sample such as blood, urine or tissue which may influence or predict the incidence of outcome or morbidity (Fathi et al., 2014). As diagnostic markers, the biomarkers could be exploited in women with positive finding to increase the precision of distinguishing BC from a benign lesion and hence preventing from unnecessary surgery (Wang et al., 2010). Reverse transcription polymerase chain reaction (RT-PCR) as a molecular technique has been used to measure tumor-specific mRNA expression. Tumor-specific mRNAs are specifically expressed and they will be markedly up regulated in tumor cells. Meanwhile, tumor markers can also be detected with various degrees of sensitivity and specificity (Gilbey et al., 2004).

**Table 1:** Pathological Characteristics of Breast Cancer Patients

Characteristic	N	%
Patients	30	100
Mean age= 48.23±2.21(range 23-87)		
<50 years	17	56.7
≥50years	13	43.3
Histological subtype		
Invasive ductal carcinoma(IDC)	17	56.7
Invasive ductal carcinoma nose type (IDC(NOS))	9	30
Other subtypes	4	13.3
Tumor size		
≤2 cm	5	16.7
>2cm	25	83.3
Lymph node status		
Negative	17	56.7
Positive	13	43.3
Histopathologic grade		
G І (well)	3	10
G II (moderate)	13	43.3
G III (poor)	11	36.7
Unknown	3	10
Stage		
І	3	10
IIA	15	50
IIB	7	23.3
III	4	13.3
IV	1	3.3
Lymphovascular Invasion (LVI)(+)	7	23.3
(-)	23	76.7
Perineural Invasion (PNI)(+)	16	53.3
(-)	14	46.7
Tissue marker status		
Ki67 +	23	76.7
Unknown	4	13.3
ER +	19	63.3
Unknown	4	13.3
PR +	17	56.7
Unknown	4	13.3
P53 +	8	26.7
Unknown	4	13.3
Her2 +	12	40
Unknown	4	13.3
Immunohistochemical profile		
Group A (HR positive and HER-2 negative)	12	40
Group B (HR positive and HER-2 positive)	7	23.3
Group C (HR negative and HER-2 positive)	5	16.7
Group D (HR negative and HER-2 negative)	2	6.7
Ag CEA serum levels		
≤2(µg/L)	7	23.3
>2(µg/L)	23	76.7

N, number of subjects; ER, estrogen receptor; PR, progesterone receptor; Her2, human epidermal growth factor receptor 2; HR, hormone receptor; HR positive, ER and/or PR positive; Ag CEA, Carcinoembryonic Antigen.

**Table 2:** Sensitivity, Specificity and Predictive Value of CEA Marker

	CEA mRNA	CEA serum	CEA mRNA and/or CEA serum	CEA mRNA combined with CEA serum
Sen %	46.70%	76.70%	93.30%	45.00%
Sen (95% CI)	28.4 % to 65.7 %	57.7% to 90.1%	77.9% to 99.2%	23.1% to 68.5%
Spe %	93.30%	90.00%	83.30%	96.70%
Spe (95% CI)	77.9 % to 99.0 %	73.5% to 97.9%	65.3% to 94.4%	82.8% to 99.9%
PPV	87.50%	88.50%	84.80%	90.00%
PPV (95% CI)	61.6 % to 98.1 %	69.8% to 97.5%	68.1% to 94.9%	55.5% to 99.7%
NPV	63.60%	79.40%	92.60%	72.50%
NPV (95% CI)	47.8 % to 77.6 %	62.1% to 91.3%	75.7% to 99.1%	56.1% to 85.4%

Sen, Sensitivity; Spe, Specificity; PPV, Positive predictive value; NPV, Negative predictive value; DA, Diagnostic accuracy; 95% CI, 95% confidence interval; CEA or CEA serum marker, signifies positive if any of the two tested markers positive; CEA combined with CEA serum marker, signifies positive if both of the two tested markers positive.

**Table 3.** Correlation between Ages, Tumor Size, Stage, Grade, Lymph Node, LVI, PNI And CEA Marker in Patients

	*P* ^1^	*P* ^2^	*P* ^3^	*P* ^4^	*P* ^5^	*P* ^6^	*P* ^7^
Ag CEA	0.07	0.17	0.84	0.17	0.36	0.7	0.13
mRNA gene expression							
CEA T	0.18	0.06	0.39	0.048٭	0.56	0.61	0.029٭
CEA B	0.13	0.19	0.74	0.31	0.96	0. 27	0.73
CEA B&T	0.13	0.11	0.6	0.027٭	0.47	0.3	0.025٭

*P¹*, age ≤50years vs. >50years; *P²*, Tumor size ≤2 cm vs. >2cm; *P³*, Stage І, II vs. III, IV;*P*^4^, grade І, II vs. III; *P*^5^, lymph node positive vs. negative; *P*^6^, LVI positive vs. negative; *P*^7^, PNI positive vs. negative; T, Tumor; B, Blood.

**Table 4:** Correlation between CEA Markers in Tissue of Patients

	*P* ^1^	*P* ^2^	*P* ^3^	*P* ^4^	*P* ^5^
Ag CEA	0.31	0.68	0.94	0.87	0.47
mRNA gene expression					
CEA T	0.14	0.53	0.69	0.94	0.19
CEA B	0.06	0.18	0.21	0.39	-
CEA B&T	0.22	0.88	0.83	0.62	0.55

*P¹*, ki67positive vs. negative; *P²*, ER positive vs. negative; *P³*, PR positive vs. negative; *P*^4^, p53 positive vs. negative; *P*^5^, Her2 positive vs. negative; T, Tumor; B, Blood

**Table 5:** Distribution of Patients in Four Groups Based on Hormone Receptor and HER-2

	Group A (HR positive and Her2 negative)		Group B (HR positive and Her2 positive)		Group C (HR negative and Her2 positive)		Group D (HR negative and Her2 negative)	
	(N=12)	%	(N=7)	%	(N=5)	%	(N=2)	%
Ag CEA (+)	9	75	6	85.71	4	80	1	50
mRNA gene expression								
CEA T(+)	6	50	2	28.57	1	20	1	50
CEA B(+)	6	50	2	28.57	4	80	1	50
CEA B&T(+)	4	33.33	2	28.57	1	20	1	50

T, Tumor; B, Blood

**Table 6:** Comparison of CEA Positive Marker between Groups

	*P* ^1^	*P* ^2^	*P* ^3^	*P* ^4^	*P* ^5^	*P* ^6^
Ag CEA (+)	0.008٭	0.043٭	0.35	0.14	0.5	0.6
mRNA gene expression						
CEA T(+)	0.05	0.19	0.19	0.6	0.59	0.72
CEA B(+)	0.15	0.026٭	0.33	0.3	0.65	0.48
CEA B&T(+)	0.1	0.28	0.28	0.53	0.53	0.68

*P¹*, Group A vs. Group B; *P²*, Group A vs. Group C; *P*^3^, Group A vs. Group D; *P*^4^, Group B vs. Group C; *P*^5^, Group B vs. Group D; *P*^6^, Group C vs. Group D; T, Tumor; B, Blood

The aim of this study is the sensitivity and specificity assessment of the carcinoembryonic antigen (CEA) by RT-PCR assay in peripheral blood (PB). CEA in breast tumor tissue was also evaluated as a diagnostic tool and the statistical correlation between the presence of CEA mRNA and clinical and pathological features was assessed in Iranian specimens. 

## Materials and Methods

*Patients and healthy control*s

30 female breast cancer (BC) patients without any neoadjuvant chemotherapy before surgery who referred to Milad hospital, Tehran, between May 2012 and March 2013, were included in this study. The patients’ ages ranged from 23 to 87 years (median=48 years). Clinical assessment was carried out based on histological reports at different stages. Our study population contained BC patients in different stages (10% in Stage I, 50 % in Stage IIA, 23.3% in Stage IIB and 17% in Stage III/ IV). BC staging (I–IV) was classified according to the standard criteria based on data of TNM (tumor, nodes and metastases) and American joint committee on cancer staging system (AJCC). The tumors were histologically graded according to the modified Bloom-Richardson grading system. Information concerning age, diagnosis and clinical pathology of each patient is shown in Table 1. 30 age-matched healthy female volunteers with no history of BC were considered as control. This study was approved by the ethical and scientific committee of institute Pasteur of Iran and written consent forms were signed by the subjects to collect their specimens.

*Bio-specimen collection*

Peripheral blood (PB) samples were collected from the patients prior to treatments and from healthy controls in 2 separate glass tubes; a buffered sodium citrate tube for RNA extraction and the one free of additive for serum collection. Serum was separated by centrifugation (2,500 rpm, 10 min) and stored at -20°C for later analysis. Fresh tissue was immediately collected after surgery and preserved in special fixative RNA later as a RNA preservative.

*Detection of CEA*

The carcinoembryonic antigen (CEA) was analyzed in serum specimens by direct sandwich technique using commercially available CanAg CEA EIA kit (FUJIREBIO Diagnostics, Inc.). When the reaction was terminated by a stop solution (0.12 M hydrochloric acid), the absorbance (optical density at 405-630nm) was measured by an ELISA reader. The standard curve was prepared based on absorbance. Values > 2 µg/l were considered as abnormal.

*RNA isolation*

AccuZol™ (BIONEER) reagent was used for isolation of total RNA from tumor tissues and blood specimens. In a typical procedure, 750µl AccuZol was added to 250µl blood sample or 50-100mg of tissue for sample lysis and denaturation followed by 5 min incubation at room temperature. Then, 0.2 ml chloroform was added to the tube; after centrifugation at 4◦C, the supernatants containing the intact RNA was transferred to a new tube, RNA was then precipitated with equal volume of isopropyl alcohol, and washed with 80% ethanol. The RNA was solubilized in RNase free water. Nanodrop and agarose gel stained with ethidium bromide were used to assess RNA quantity and integrity, respectively. After ensuring on the quality of purified RNA, it was used for gene expression. 

*Reverse Transcriptase -Polymerase Chain Reactions (RT-PCR)*

Total extracted RNA was amplified by one step AccuPower® RT-PCR Premix (BioNEER) Kit. All of the required components of cDNA synthesis and amplification were provided in one tube. The applied GAPDH and CEA-specific primers are shown below: GAPDH sense: 5’-GGTCGGAGTCAACGGATTTG-3’and antisense: 5’-ATGAGCCCCAGCCTTCTCCAT-3’. CEA sense: 5’-GGGCCACTGTCGGCATCATGATTGG-3’and antisense: 5’-TGTAGCTGTTGCAAATGCTTTAAG GAAGAAGC-3’.

The quality of RNA isolates was verified by amplification of GAPDH (glyceraldehyde-3-phosphate dehydrogenase). Presence of GAPDH mRNA (31.2pg) was considered as an internal control. The purpose of the internal control gene GAPDH was to normalize the PCRs for the amount of RNA added to the reverse transcription reactions. In this experiment, extracted RNA (1-2μg) was used with the same amount of GAPDH RT-PCR product to obtain the same sensitivity for CEA marker gene expression. The extracted RNA and the reverse primer were mixed and incubated at 70ºC for 5 min. The incubated mixture and the forward primer were then transferred into a premix tube. Synthesis of cDNA was performed at 42ºC for 60 min and at 94ºC for 5 min. 30-cycle PCR was found optimal. PCR cycles were carried out as follows: 94ºC for 60 sec, 54ºC for 30 sec and 72ºC for 60 sec with a final 10-min extension at 72ºC.The aliquots of PCR products were visualized by electrophoresis on 2% agarose gel under UV. As a positive control, we used 1-2μg of total RNA from the human breast cancer cell line MCF-7; sterile water was also used instead of RNA as a negative control for RT-PCR.

*Statistical Analysis*

MedCalc. Statistical software was used to compute sensitivity, specificity, positive predictive value (PPV) and negative predictive value (NPV). Pearson’s chi-squared test was performed to evaluate the correlation between CEA mRNA in PB or tumor tissue and the age, tumor size, stage, histological grade, lymph node status, tissue marker status, lymphovascular invasion (LVI), perineural invasion (PNI) and serum CEA. P-values below 0.05 were considered statistically significant. The analyses were performed using SPSS software version 18.

## Results

*Diagnosis sensitivity and specificity*

RT-PCR analysis of CEA were detected in 14 PB (46.7 %) and 11 (36.7%) tissue of BC patients. CEA mRNA was also expressed in 2 PB (6.7 %) of healthy female subjects. Expression of CEA mRNA significantly differ (P<0.001) from the controls. In BC patients, CEA mRNA were positive in 9 (30.0 %) and negative in 14 (46.7%) of PB and tissue samples. 

In Table 2 the sensitivity, specificity, positive predictive value (PPV) and negative predictive value (NPV) of CEA mRNA alone and its combination with serum CEA was illustrated in 30 BC patients. 46.7 % (95% CI = 28.4 % to 65.7 %) sensitivity, as well as 93.3% (95% CI =77.9 % to 99.0 %) specificity was shown for CEA mRNA. The positive and negative predictive rates for CEA mRNA were 87.5 % (95% CI = 61.6 % to 98.1 %) and 63.6 % (95% CI = 47.8 % to 77.6 %), respectively. The sensitivity and specificity of serum CEA for detection of malignant BC disease was 76.7% (95% CI = 57.7% to 90.1%) and 90.0% (95% CI =73.5% to 97.9%), respectively; where 2µg/L was considered as the cut-off level. The positive and negative predictive rates were 88.5% (95% CI =69.8% to 97.5%) and 79.4 %( 95% CI =62.1% to 91.3%). Sensitivity, specificity, positive and negative predictive rates were 93.3% (95% CI = 77.9% to 99.2%), 83.3 % (95% CI = 65.3% to 94.36%), 84.8% (95% CI = 68.1% to 94.9%) and 92.6 % (95% CI = 75.7% to 99.1%), for CEA mRNA and or CEA in serum, respectively. Combined analysis of CEA mRNA and CEA in serum showed that sensitivity, specificity, positive and negative predictive rates were 45% (95% CI = 23.1% to 68.5%), 96.7 % (95% CI = 82.8% to 99.9%), 90% (95% CI = 55.5% to 99.7%) and 72.5 % (95% CI = 56.1% to 85.4%), respectively. 

Based on Table 3, no correlation was found between CEA serum levels or CEA mRNA and the patient’s age, tumor size and stages. No significant association was observed between CEA serum levels and tumor grade. However, there was a correlation between CEA mRNA in tissue samples or its expression in both PB and tissue samples and tumor grade (p=0.048 and p=0.027, respectively). The correlation between lymph node involvement and CEA mRNA in PB or tissue samples was not significantly different. We also did not find a significant difference between CEA serum levels of positive and negative lymph node patients. CEA mRNA in tissue samples and its expression in PB and tissue samples were significantly correlated with perineural invasion (PNI) (p=0.029 and p=0.025, respectively). No correlation was found between CEA mRNA and lymphovascular invasion (LVI). We also did not find significant difference between CEA serum levels of positive and negative LVI and PNI patients.

Ki-67, estrogen receptor (ER), progesterone receptor (PR), P53 and human epidermal growth factor receptor 2 (HER2) of 26 BC patients were available. CEA serum levels and CEA mRNA in PB or tissue samples were compared with proliferation marker Ki-67, ER, PR, P53 tumor suppressor protein and HER2 which no significant difference was shown (Table 4). 

Based on HR and HER-2, BC patients were separated into four groups (Table 5) to compare the positive levels of CEA mRNA and CEA serum between distinct groups. Patients who were HR positive (ER and PR positive) and HER-2 negative were included in group A (12 patients, 46.1%). Patients who were HR positive and HER-2 positive were in group B (7 patients, 26.9%). Patients with HR negative (ER and PR negative) and HER-2 positive were in group C (5 patients, 19.2%); while Group D included patients with HR negative and HER-2 negative (2 patients, 7.7 %).

In Table 6, there was a significant difference in CEA mRNA positive rates of PB samples in group A vs C (p=0.027); moreover, a significant difference was observed in the CEA serum positive rates of group A compared with B and C (p=0.008 and p=0.043, respectively). There was no significant difference between other groups in terms of CEA mRNA and CEA in serum.

## Discussion

In order to evaluate the biomarkers potential in BC detection, we analyzed the expression of CEA mRNA in the PB of BC patients and normal subjects by RT-PCR method. While CEA mRNA was observed in a number of normal subjects, but the results of our study demonstrate a significant difference between normal subjects and BC patients in terms of tested CEA tumor marker. According to some studies, CEA mRNA was not detected by RT-PCR in the PB of normal females (Stathopoulou et al., 2003; Gerhard et al., 1994; Mori et al., 1998). However, CEA mRNA may be a reliable marker for the detection of occult BC cells contamination. Our data is also supported from an earlier report (Corradini et al., 2001). 

Basically, a diagnostic biomarker should have high sensitivity and specificity (Frantzi et al., 2014). Analysis of CEA mRNA detection in patients revealed that this mRNA blood test is not reliable for BC diagnosis especially due to the lack of sensitivity (46.7%); it may miss cancers even with a high specificity (93.3 %). These results are comparable to our previous reports indicating that CEA mRNA was positive 71.7% and 6.7% in PB of 60 BC patients and 30 healthy subjects, respectively (Oloomi et al., 2013).

In this study, the detected CEA mRNA from the BC tissue specimens did not show significant difference with PB. In addition, there was a high direct correlation between the expression of CEA mRNA in PB and tissue samples (Pearson chi-square = 8.623, P=0.003). Hence, RT-PCR of CEA was almost sensitive to PB and tissue specimens of BC patients. Conversely, it has been reported (Ghaffari et al., 2006) that CEA mRNA showed higher sensitivity (95.0 %) in tissues.

In clinical practice, CEA serum levels are commonly used to manage the colorectal cancer patients (Saito et al., 2017). CEA in the serum is not a colorectal carcinoma-specific antigen and its level may increase in other cancers such as BC and it has also been established as a prognostic marker in BC patients (Li et al., 2018). In our study, serum from female BC patients was also examined for the presence of the CEA by direct ELISA method. Similar to our pervious study, it was notably elevated in BC patients compared to normal (1.8±0.1 µg/L, 6.0±0.5 µg/L, respectively, p=0.00) (Moazzezy et al., 2014). Higher levels of CEA positive in serum was compared to CEA mRNA positive in PB samples of BC (p=0.03), with only 10% false positive results in healthy controls. However, Molina et al. reported that the preoperative sensitivities of CEA in serum were 11.7%–13.0% which raised to 30.0%–70.0% after recurrence in locoregional BC patients (Molina et al., 2016). It should be noted that prognosis of patients whose serum CEA level was within the normal range at the time of diagnosis is significantly better than those with elevated CEA levels (Uehara et al., 2008).

Among the investigated molecular markers, CEA mRNA in tumoral tissue samples and CEA mRNA in both PB and tissue samples were significantly correlated with serum CEA (Pearson chi-square =-4.751, p=0.029 and Pearson chi-square =-7.462, p=0.006, respectively).

The mRNA detection has already been limited based on single marker (Yu et al., 2013). Therefore, we assessed both CEA mRNA and serum in PB of female BC patients. However, the rate of BC detection for CEA serum combination with CEA mRNA was 45.0%. Nonetheless, CEA mRNA in PB combined with CEA serum showed the highest specificity, with only 3.0 % false positive results in healthy females. As we know, while the specificity of each marker may be low, the combination of different suitable tumor markers can be a potent clinical tool (Oloomi et al., 2018).

Generally, RT-PCR specificity is higher than ELISA. ELISA is more sensitive but not specific. In our study, we used combined two techniques; RT-PCR and ELISA. Both two techniques exhibited a low sensitivity but a high specificity for detection of CEA marker the same as using RT-PCR technique, alone. The ELISA detection system for CEA is more sensitive than the RT-PCR but the specificity was the same. Our results in CEA and/or CEA serum marker group were shown the most sensitivity. Based on our results, the RT-PCR technique alone cannot play an efficient role as diagnostic technique, although the small number of samples used in this study can be effective in this result.

The purpose of clinical and pathological parameters study is to provide the best decisions about effective management of BC (Patani et al., 2018). Therefore, we assessed the correlation of CEA mRNA positivity in the PB or tissue with traditional prognostic factors such as age, tumor size, stage and grade, nodal status, lymphovascular invasion (LVI) and perineural invasion (PNI) in BC patients. Some researchers reported that deregulated overexpression of multiple CEA markers, such as CEA are able to block cellular differentiation in large number of cell types; and CEA were found to be overexpressed in BC tissue specimens in high grade tumors (Michaelidou et al., 2013, Ilantzis et al., 2002). Unexpectedly in our results, CEA mRNA in BC tissue specimens and CEA mRNA expression in both PB and tissue specimens were correlated significantly with grade I/II (Pearson chi-square = -3.91, p=0.048 and Pearson chi-square = -4.90, p=0.027, respectively), when analyzed between low and intermediate grades I/II and poorly differentiated histopathological grade III tumors. This discrepancy may be due to the small sample size of our study. 

Concerning the prevalence of CEA in lymphovascular invasion (LVI) positivity, the comparison was not statistically significant for CEA mRNA. The presence of LVI is an independent significant prognostic factor in BC lymph node-positive patients (with metastasis to axillary lymph node) as well as lymph node-negative patients (without metastasis to lymph nodes) (Song et al., 2011). In our study, correlation between CEA in lymph node positive or negative groups and LVI was not significant (data was not shown). Notably, LVI should not by itself be considered sufficient to move BC patients from a low-risk group to a high-risk one and is not an independent risk factor for a poorer prognosis (Ejlertsen et al., 2009). Based on our data, perineural invasion (PNI) showed inverse correlation with CEA mRNA in tissue and in both PB, and tissue samples (Pearson chi-square=-4.74, p=0.029 and Pearson chi-square=-5.00, p=0.025, respectively). PNI is a less common trait in BC tissue and has no prognostic importance compared to the vascular invasion (VI) (Duraker et al., 2006). However, VI did not show any correlation in our study (Data was not shown). It has been also reported (Karak et al., 2010) that PNI was a partly infrequent histologic particularity in invasive breast cancer happening 10 times less than LVI.

A limited number of studies have addressed the correlation between CEA and molecular subtypes (Shao et al., 2015). Based on the immunohistochemical assessment of ER, PR, HER2, p53 and Ki57, a panel of markers plays a necessary role in the individualized care of BC (Aguiar et al., 2013). No correlation was observed between CEA mRNA, serum and immunohistochemical parameters. However, Lee et al. reported that CEA was positively correlated with HER2 expression, but not related to ER or PR status in breast cancer (Lee et al., 2013).

In our study, 26 invasive breast cancers (IBC) patients were classified based on hormone receptors (ER/PR) and human epidermal growth factor 2 (HER2). They were divided into four groups. If the breast cancer (BC) cells contain either ER or PR receptors, they called hormone receptor-positive (group A). Triple-positive was used to describe cancers cases that were ER-positive, PR-positive and had too much HER2 (group B). HER2 positive and high levels of HER2 were used to describe group C. Finally, if the BC didn’t have estrogen (ER) or progesterone (PR) receptors and didn’t have too much HER2, they were called triple negative (group D). The levels of CEA mRNA and serum were compared between the different molecular subtypes groups. After comparing CEA mRNA in PB of different groups, only the HER-2 negative tumors with positive hormone receptors (HR) (group A) were significantly different from HER-2 positive tumor group that are hormone receptors (HR) negative (group C) (Pearson chi-square=-4.95, p=0.026). Based on comparing the positive rates of CEA serum in different groups, HER-2 negative and HR positive tumors (group A) were significantly different from triple positive tumors (group B) and HER-2 positive and HR negative tumors (group C) (Pearson chi-square=-7.01, p=0.008 and Pearson chi-square=-4.09, p=0.043). Notably, HER-2 positive tumors that are hormone receptors (HR) negative (group C) are traditionally associated with poor prognosis (Yan et al., 2015). Wu SG et al. reported that CEA serum levels were higher in patients with HER2 positive BC and lower in those with triple-negative BC as undifferentiated subtype which justifies that different molecular subtypes have different biological behaviors (Wu et al., 2014). 

In conclusion, high level expression of glycoprotein carcinoembryonic antigen (CEA) can directly signal the cancer. Still, simple and high detection sensitivity of CEA is of great significance. An established tumor marker such as CEA is valuable in late stages and support therapy response assessment and early detection of recurrent disease. In early stages, their sensitivity is limited. One of the most serious issues in the research and evolution of tumor markers is still the improvement of sensitivity and specificity. Nowadays, focus on combined biomarker approach shows improved sensitivity for the breast cancer detection (Zaleski et al., 2018).

It concluded that the diagnostic value of CEA mRNA combined with CEA serum has the sensitivity and specificity in breast cancer. Additionally, CEA mRNA has a diagnostic value, since the expression levels of this maker in PB was the same as tumoral tissues. Therefore, estimation of CEA in serum with quantitative CEA mRNA is a good strategy for detection of primary breast cancer. It should be noted that the prognostic value of our data requires follow-up studies on a larger group of patients, in future.
